# Effect of Microwave Treatment of Graphite on the Electrical Conductivity and Electrochemical Properties of Polyaniline/Graphene Oxide Composites

**DOI:** 10.3390/polym8110399

**Published:** 2016-11-16

**Authors:** Yanjun Tang, Xiulan Hu, Dongdong Liu, Daliang Guo, Junhua Zhang

**Affiliations:** 1 National Engineering Laboratory of Textile Fiber Materials and Processing Technology, Zhejiang Sci-Tech University, Hangzhou 310018, China; selene1225@163.com (X.H.); adoreguy@163.com (D.L.); guodl@zstu.edu.cn (D.G.); zhangjh@zstu.edu.cn (J.Z.); 2 Key Laboratory of Recycling and Eco-treatment of Waste Biomass of Zhejiang Province, Zhejiang University of Science and Technology, Hangzhou 310023, China; 3 Key Lab of Pulp and Paper Science & Technology of Ministry of Education, Qilu University of Technology, Jinan 250353, China; 4 Key Lab of Biomass Energy and Material, Nanjing 210000, China

**Keywords:** conducting polymers, nanostructured polymers, surfaces and interfaces, electrical conductivity, electrochemical properties

## Abstract

Polyaniline (PANI)/graphene oxide (GO) composites were synthesized via in situ polymerization of aniline in the presence of GO. The effect of microwave treatment of graphite on the electrical conductivity and electrochemical properties of PANI/GO composites was highlighted, and the morphology and microstructure were subsequently characterized using transmission electron microscopy, scanning electron microscopy, Fourier-transformed infrared spectroscopy, X-ray diffraction, and thermogravimetric analysis. The results demonstrated that microwave treatment of graphite imparted a well-dispersed, highly ordered layered structure to the as-prepared GO, and in turn facilitated strong bonding between the GO and PANI nanosheets, which may be responsible for the improved electrical conductivity and electrochemical properties of the resulting PANI/GO composites. The desired PANI/GO composites possessed an electrical conductivity of 508 S/m, an areal capacitance of 172.8 mF/cm^2^, and a retained capacitance of 87.4% after cycling, representing percentage increases of 102, 232, and 112, respectively, as a result of the microwave treatment of graphite. The resulting composites are promising electrode materials for high-performance and ecofriendly electrical energy storage devices.

## 1. Introduction

In recent years, there has been an ever-increasing demand for environmentally friendly and high-performance energy storage devices [1]. Among the various energy storage devices, supercapacitors (SCs) have received considerable attention due to their high power density and extremely long cycle life [2]. However, the energy density of commercially available supercapacitors is still lower than those of batteries and fuel cells [3]. Therefore, there is increasing pressure to improve the electrochemical properties of SCs while maintaining their intrinsic high power density. Intrinsically conducting polymers (ICPs) [4] (e.g., polyaniline (PANI) [5], polypyrrole (PPy) [6], and polythiophenes (PT) [7]) have shown promise for application as electrode materials for SCs due to their strategic flexibility and considerable specific capacitance [8]. Among these ICPs, PANI has been regarded as one of the best candidates for electrode materials for its high capacitive characteristics, low cost, and ease of synthesis [9,10]. However, the relatively poor cycling life imposes considerable restrictions on its practical applications [11]. Graphene oxide (GO), as an important graphene derivative, has a larger specific surface area, a wide chemical potential, excellent chemical stability, and a richly draped morphology, thus holding great promise for energy storage applications [12,13]. Therefore, the combination of GO and PANI might be expected to exert a synergistic effect in improving the overall properties of PANI/GO composites [14,15]. In fact, the preparation and application of PANI/GO composites is of great interest to academic and industrial organizations [16]. For instance, Bissessur et al. [17] reported that PANI can be directly inserted into GO without preparing precursor intercalation compounds by taking advantage of the exfoliation properties of the layered host. Wang et al. [18] systematically investigated the influence of raw material sizes and feeding ratios on the electrochemical capacitance and chemical structures of GO-doped composites. In addition, Meriga et al. [19] proposed a novel method to produce a combination of sulfonated PANI (SPANI) and reduced graphene oxide (RGO); the resulting SPANI/RGO composite exhibited superior thermal stability and electrochemical properties as compared to pure PANI.

In general, the presence of GO in PANI/GO composites may provide the following principal benefits [20]: (1) an increase in the specific surface area of composites due to GO serving as an “anchor” for PANI indifferent forms; (2) a decrease in the mechanical deformation of PANI and composites during the charge transport process because of the mechanical stability of graphene; and (3) fast transportation of charge carriers through the electrode matrix facilitated by easy conductive channels arising from the good interfacial interaction between GO and PANI. In this regard, it is essential to enhance both the distribution of graphene layers in the polymer matrix and the interfacial bonding between the GO layers and polymer matrix. In this context, great effort has recently been dedicated to surface modifying GO and/or optimizing the preparation procedures for further enhancing the synergistic effect of nanofillers and the polymer matrix [21,22]. For instance, Ke et al. [23] prepared GO/PPy/CdSe nanocomposites with electrochemiluminescence (ECL) properties using a novel and simple method, and found that the desired ECL properties were strongly dependent on the synergistic effect of GO and PPy. Xu et al. [24] introduced a facile method to construct hierarchical nanocomposites, wherein aligned PANI nanowires were carefully synthesized on two-dimensional GO nanosheets by dilute polymerization. In addition, Kumar et al. [25] produced highly conducting PANI-grafted reduced graphene oxide (rGO) composites with enhanced properties using a new, facile route by functionalizing graphite oxide with 4-aminophenol via acyl chemistry.

Microwaves are a form of electromagnetic energy, and microwave irradiation is a well-known method for modifying the chemical, structural, electrical, mechanical, and other desired properties of polymer-based composites [26]. In particular, the application of microwave irradiation as an assisted role in the synthesis of expanded graphite (EG) has been widely reported. For instance, Zhang et al. [27] proposed a rapid and efficient method for the preparation of EG. The exfoliation process was accelerated by microwave irradiation, and the preparation time was greatly shortened. The obtained EG was wormlike in shape and exhibit well-exfoliated structure. Wei et al. [28] successfully prepared exfoliated graphite materials using microwave irradiation in a short time (about 4 min, including 3 min mixing and 1 min microwave irradiation). The promotion of the intercalation by microwave irradiation was proven by X-ray diffraction. Furthermore, Kwon et al. [29] fabricated the exfoliated graphite by microwave treatment for the synthesis of graphite-SO_3_ intercalation compounds (GICs). GIC was prepared by direct reaction of flake-exfoliated graphite with SO_3_ gas. More importantly, the microwave treatment was found to be able to impart the graphite felt with more hydrophilic groups, such as –OH, on its defects, excellent electrochemical activity, and increased roughness degree of the surface, which helped to increase a specific area to a great extent and improve the degrees of surface energy [30]. Overall, these changes, as a function of the applied microwave treatment, should be advantageous in facilitating the exfoliation process, oxidation reaction of graphite materials, thereby leading to the highly efficient targeted synthesis of polymer-based composites. GO is a well-known layer-structured compound obtained by oxidation of graphite. In principle, microwave treatment of graphite may at least offer several advantages over traditional approaches for the conversion of graphite to GO, which mainly includes the following aspects: (1) highly oxidized GO, especially synthesized from natural graphite with high crystallinity, can be easily dispersed; and (2) abundant functionalities on the surface of GO may create more opportunities for interfacial interaction between GO and polymeric matrix. However, to date, the effect of microwave treatment of graphite on the electrical conductivity and electrochemical properties of the resulting PANI/GO composites remains elusive. In the present work, a microwave treatment was applied to optimize the oxidation of graphite to GO, and the treatment’s effect on the electrical conductivity and electrochemical properties of PANI/GO composites prepared via in situ polymerization was studied. The microstructure and crystal structure of the resulting composites were characterized by transmission electron microscopy (TEM), scanning electron microscopy (SEM), Fourier-transformed infrared spectroscopy (FT-IR), X-ray diffraction (XRD), and thermogravimetric analysis (TGA). This work represents a large step forward on the way to developing polymer/GO composites for electrochemical energy storage devices with a high energy/power density and highly efficient environmental remediation.

## 2. Materials and Methods

### 2.1. Materials

Natural graphite flake (500 mesh) was kindly provided by Hangzhou Xinhua Paper Co., Ltd. in Hangzhou, China. Aniline (An, 99%, *w*/*w*), ammonium persulfate (APS), doping acid hydrochloric acid (HCl), sulfuric acid (H_2_SO_4_, 98%, *w*/*w*), and hydrogen peroxide (H_2_O_2_, 30%, *w*/*w*) were purchased from Hangzhou Mick Chemical Instrument Co., Ltd. (Hangzhou, China). The An was stored in a refrigerator prior to use. A perfluorosulfonic acid–PTFE (polytetrafluoroethene) copolymer (5%, *w*/*w*) solution was purchased from Alfa Aesar (Shanghai, China). All the reagents were analytical grade and used without further purification. Distilled water was used for all experiments.

### 2.2. Microwave Treatment of Graphite

Raw graphite particles were treated in a microwave oven (Haier/MF-2270MG, Haier Electronics Group Co., Ltd., Qingdao, China) at a frequency of 2.45 GHz. The microwave oven had a maximum power of 700 W with six discrete settings. The raw graphite was placed in a sealed glass vessel that was permeable to microwaves. Upon microwave irradiation, a large volume expansion of the raw graphite powders accompanied by violent burning was observed instantaneously at medium power (380 W) and in less than 40 s at low power (120 W). Consequently, the graphite was subjected to microwave irradiation for 30 s at 120 W, and the treated graphite was named M-graphite.

### 2.3. Synthesis of M-GO

**Figure 1:**
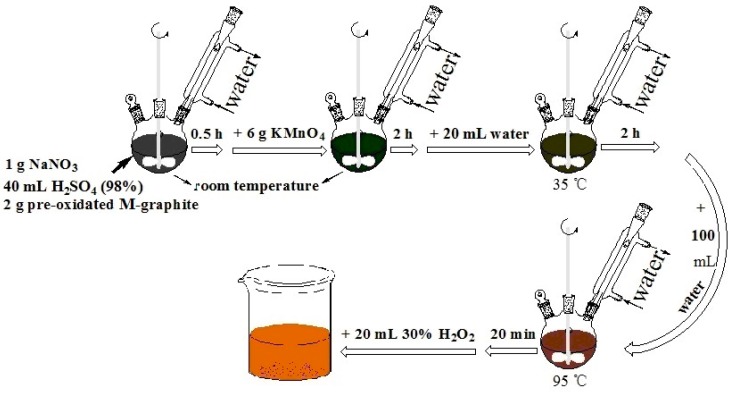
A process for graphene oxide production.

A modified Hummers’ method was used to prepare graphene oxide (GO) [31]. Pre-oxidation was carried out on the M-graphite prior to the regular oxidation. Initially, M-graphite (2 g) and Na_2_S_2_O_8_ (2 g) were mixed with 20 mL concentrated sulfuric acid (H_2_SO_4_, 98%, *w*/*w*) in a 100 mL three-neck round-bottom flask. The mixture was stirred at 80 °C for 6 h during a complete condensation cycle to obtain pre-oxidated graphite. Then, concentrated sulfuric acid (40 mL) was transferred to a 250 mL three-neck round-bottom flask, and pre-oxidated M-graphite (2 g) and NaNO_3_ (1 g) were simultaneously added into the sulfuric acid solution and stirred for 30 min at room temperature, followed by adding KMnO_4_ (6 g) while ensuring the temperature stayed below 20 °C. The reaction was run at room temperature for 2 h, and distilled water (20 mL) was then slowly added to the flask. Afterwards, the mixture was stirred for 2 h at 35 °C. The mixture was further diluted with 100 mL distilled water, and the reaction continued for 15 min at 95 °C, followed by the addition of 20 mL H_2_O_2_ (30%, *w*/*w*). The resultant mixture was centrifuged at 9000 rpm for 10 min, and the sediment was repetitively washed with distilled water and 1 M HCl until the pH was approximately 7. The resulting homogenized sediment was ultrasonicated for 30 min and then dried at 60 °C for 24 h in a vacuum oven. Eventually, the targeted product was obtained and named as M-GO. The synthesis process for M-GO is shown in Figure 1.

### 2.4. Synthesis of PANI/M-GO Composites

PANI/M-GO composites were obtained with different mass ratios by in situ polymerization in the presence of M-GO and aniline monomer, as recorded elsewhere. A typical synthesis procedure is as follows, taking aniline/M-GO with a mass ratio of 10:1 as an example. First, 0.1 g M-GO was dispersed into 50 mL distilled water, and 1 mL aniline was added quickly to avoid being oxidized by air. Then, the solution was ultrasonicated for 30 min to form a stable aniline/M-GO mixture. Under violent stirring at room temperature, an APS aqueous solution was added to the above solution to achieve an APS/aniline mole ratio of 1:4 in 100 mL 1 M HCl [32]. The chemical polymerization was then performed overnight at room temperature. Afterwards, the composite was filtered and repetitively washed with water and ethanol. Eventually, the obtained sample was dried at 60 °C for 24 h. The resulting product was collected and labeled as the appropriate PANI/M-GO composite. For comparison, GO and PANI/GO composites were also prepared from raw graphite without the microwave treatment via the same procedure.

### 2.5. Electrical Conductivity Measurements

The electrical conductivities of pressed pellets of PANI/GO or PANI/M-GO composites were measured by a digital four-probe tester (SZT-2, Suzhou Tongchuang Electronics, Suzhou, China).

### 2.6. Electrochemical Measurements

Each PANI/M-GO composite sample was mixed with the perfluorosulfonic acid–PTFE copolymer (5% *w*/*w* solution) to form a slurry, which was then deposited on a glassy carbon electrode (3 mm diameter, BAS). The as-prepared electrode was allowed to dry in air prior to the electrochemical characterization.

All cyclic voltammetry (CV) measurements were carried out in a 1 M sulfuric acid aqueous solution using a three-electrode system, with a calomel electrode (Hg/Hg_2_Cl_2_) as the reference electrode, a platinum electrode (Pt) as the counter electrode, and the as-coated glassy carbon electrode as the working electrode.

### 2.7. Characterizations and Analysis

SEM (SU8000, Hitachi, Japan) was used to investigate the morphology of the samples. The sample powders were spread onto conducting adhesive, and the images were acquired at an accelerating voltage of 5 kV. All samples were prepared by drying a drop of a sample/water suspension on the carbon-coated copper grid under an infrared lamp for 5 min, and images were acquired using TEM (JSM-2100, JEOL, Tokyo, Japan) at an accelerating voltage of 200 kV. FT-IR spectra for pure PANI, GO, PANI/GO, and PANI/M-GO composites were recorded on a Nicolet 5700 spectrometer (Thermo Fisher Scientific, Waltham, MA, USA) at 1 cm^−1^ resolution and 20 scans per spectrum on pellets of KBr. XRD analysis was carried out on a diffractometer (XʹTRA–055, ARL, Basel, Switzerland) with a nickel-filter Cu Kα radiation source (λ = 0.154 nm). XRD scans were recorded from 3° to 60° for 2θ with a speed of 3°/min. The thermal stability of all samples was determined using Pyrisl TGA (Perkin Elmer, Waltham, MA, USA) under a nitrogen atmosphere from 25 to 800 °C at a heating rate of 10 °C/min.

## 3. Results and Discussion

### 3.1. Effect of the Microwave Treatment of Graphite and Feeding Ratio on the Electrical Conductivity of PANI/GO Composites

**Figure 2:**
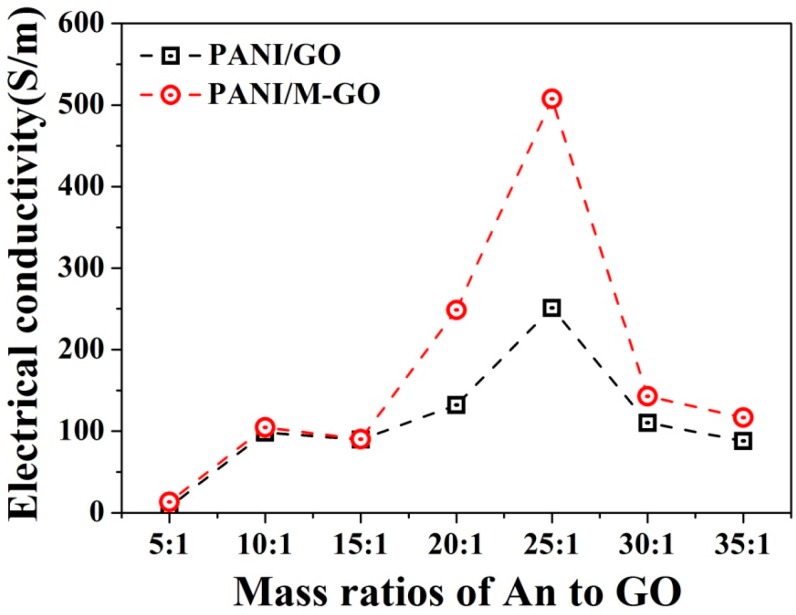
Electrical conductivities of PANI/GO composites as a function of microwave pretreatment of graphite and different feeding ratios.

The feeding ratio is one of the most important controlling factors determining the electrochemical properties of the resulting composites via in situ polymerization. For this reason, the optimum An/GO mass ratio was defined to achieve the desired electrical conductivity of the PANI/GO composites. The electrical conductivities of the PANI/GO composites were measured as a function of the An/GO mass ratio. The results in Figure 2 show that various An/GO mass ratios conferred different electrical conductivities to the PANI/GO composites. At a mass ratio of 5:1, the composites exhibited an electrical conductivity of 7.3 S/m, which continuously increased with the increasing mass ratio of up to 25:1, mainly due to the presence of PANI derived from in situ polymerization. However, a pronounced downtrend in electrical conductivity was observed when the mass ratio further increased to 35:1. This result provides direct evidence that there is a proper range of feeding ratios that enables improvement of the electrical conductivity of PANI/GO composites. Actually, a similar observation was reported earlier by Zhao et al. [33]. In that paper, the influence of GO on the electrical conductivity of PANI/GO composites was investigated and explained. In the current work, the electrical conductivity reached the maximum value when An/GO mass ratio was located at 25:1. This unusual and unexpected finding may be mainly attributed to the template effect of graphene oxide induced by the exfoliation of GO. In principle, in the presence of graphene oxide, the conductive PANI exhibits a preferably oriented structure, and the oriented polymer chains allow the carrier to move easily [34]. In this regard, higher GO content may be prone to offer more templates for PANI. Nevertheless, once the GO content exceeds a certain value, a sharp decrease in electrical conductivity would inevitably been countered due to the insulation of GO.

Besides the feeding ratio, the effect of microwave treatment of graphite on the electrical conductivity of PANI/GO composites was also studied. Note that PANI/M-GO composites exhibited higher electrical conductivity than that of PANI/GO composites. In particular, at an An/M-GO mass ratio of 25:1, the electrical conductivity of the resulting composites increased by 102% due to the microwave-treated graphite. The improved electrical conductivity induced by microwave treatment of graphite may be attributed to the fact that microwave energy is prone to impart a highly ordered layered structure to GO, thus eventually facilitating the fabrication of PANI/M-GO composites [35]. As stated above, both the feeding ratio and the microwave treatment of graphite have an important effect on the electrical conductivity of PANI/GO composites. As a result, the microwave treatment and the mass ratio of 25:1 for An/GO were thereafter used to prepare the PANI/GO composites in the subsequent experiments.

### 3.2. Effect of the Microwave Treatment of Graphite on the CV Curves of the PANI/GO Composites

**Figure 3:**
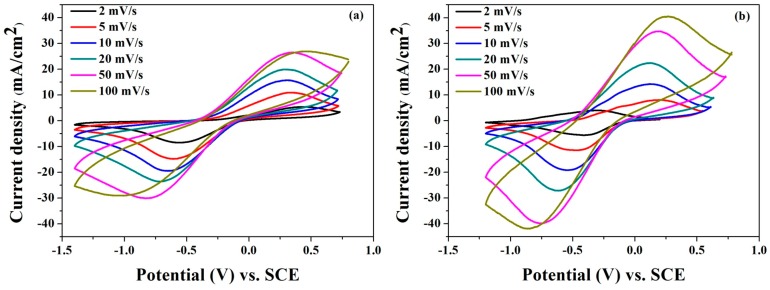
Cyclic voltammetry (CV) curves of (**a**) polyaniline (PANI)/GO and (**b**) PANI/M-GO composite electrodes at different scan rates in 1 M H_2_SO_4_.

CV is often used as a diagnostic tool for elucidating electrode mechanisms [36]. The CV curves of the PANI/GO and PANI/M-GO composites recorded at different scan rates in 1 M H_2_SO_4_ are shown in Figure 3a,b, respectively. The figures show that the oxidation peaks right-shifted to higher voltages, and the reduction peaks shifted to lower values with the increasing scan rate from 2 to 100 mV/s, mainly due to the resistance of the electrode [37]. On the other hand, as the scan rate increased, there was a linear increase in the current density within the entire potential range. This effect was more evident for the lower scan rate, indicating a good rate capability for PANI/GO and PANI/M-GO composite electrodes.

**Figure 4:**
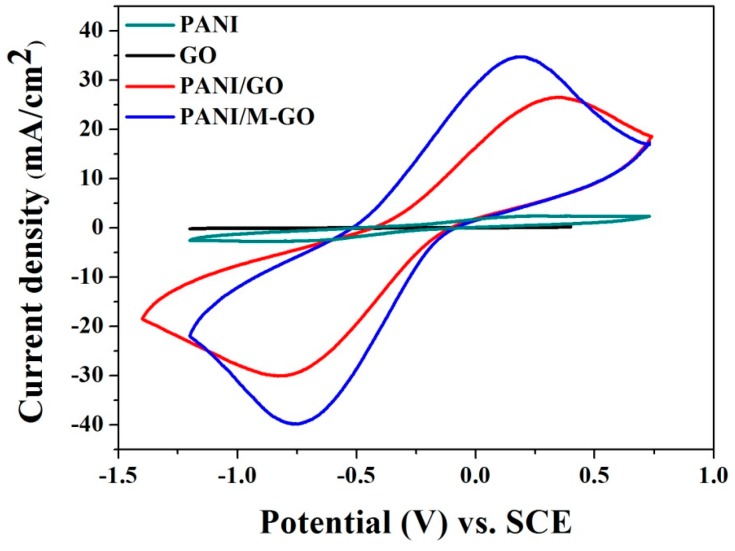
CV curves of PANI, GO, PANI/GO, and PANI/M-GO composite electrode in 1 M H_2_SO_4_ at 50 mV/s.

Figure 4 illustrates the CV curves of pure PANI, GO, PANI/GO, and PANI/M-GO composite electrodes at 50 mV/s. The GO electrode exhibited a pair of peaks largely due to the oxygen-containing groups around the surface. Meanwhile, the CV curve of GO electrode was nearly rectangular, which indicates that the GO electrode had good charge propagation but low conductivity. A pair of redox peaks in the CV curve of the PANI electrode was observed, which correspond to the redox couple transition between the leucoemeraldine form (semiconducting state) and the polaronicemeraldine form (conducting state) and resulted in the redox capacitance [38]. Compared with the GO and PANI electrodes, the composite electrodes exhibited superior current density responses, which signifies the improved electrochemical performances of the composites due to the synergistic effect of GO and PANI. Furthermore, note that the electrochemical capacitance of PANI/M-GO composite electrode was better than that of the PANI/GO composite electrode.

### 3.3. Effect of the Microwave Treatment of Graphite on the Cycle Stability of the PANI/GO Composites

Cycle stability is a critical parameter in relation to the performance of SCs. Multiple CV curves for the composite electrodes were recorded in 1 M H_2_SO_4_ at 100 mV/s for 1200 cycles. The areal capacitance *Ca* (mF/cm^2^) of the composite was calculated from the following equation [39]:

(1) $$Ca=\frac{A}{2\nu \Delta E}$$

where *A* is the area of the specific CV curve obtained by integrating the whole CV curve, ν is the scan rate (V/s), and Δ*E* is the unidirectional voltage window (V) for the cyclic voltammetry process.

**Figure 5:**
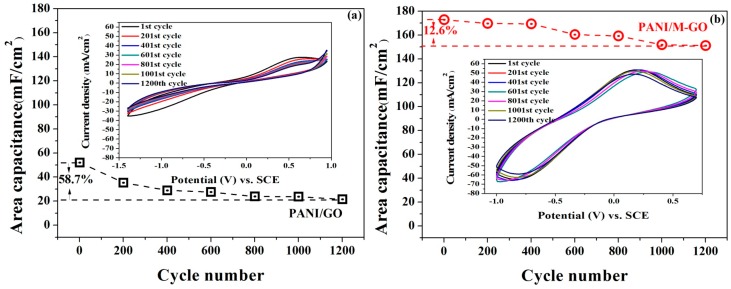
Cycle stability of (**a**) PANI/GO and (**b**) PANI/M-GO composite electrode during the multiple CVs process.

The variation of the areal capacitance of the as-prepared composites is shown in Figure 5a,b. Note that the capacitance of the PANI/M-GO composite electrode was 172.8 mF/cm^2^, which was 3.3 times higher than that of the PANI/GO composite electrode (52.1 mF/cm^2^). After 1200 cycles, over 87.4% capacitance was maintained for the PANI/M-GO composite electrode, which was much higher than that of the PANI/GO composite electrode (41.3%).

Therefore, the above results supported the conclusion that microwave treatment of raw graphite could significantly improve the electrical conductivity and electrochemical properties of the as-prepared PANI/GO composites.

### 3.4. Effect of the Microwave Treatment of Graphite on the Microstructure of PANI/GO Composites

#### 3.4.1. TEM Observations

**Figure 6:**
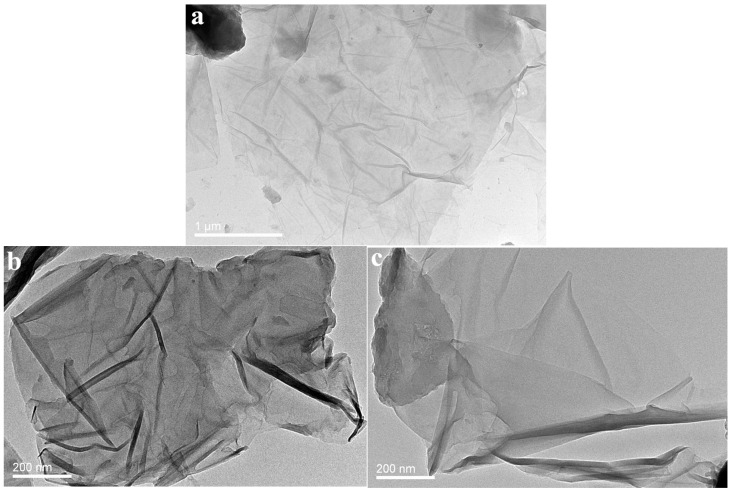
TEM images of (**a**) GO; (**b**) PANI/GO and (**c**) PANI/M-GO composites.

Typical TEM images of the GO, PANI/GO, and PANI/M-GO composites are shown in Figure 6. As can be seen in Figure 6a, GO exhibited a thin, transparent, layer-like structure with a typical folding nature, presumably due to the fact that partially conjugated structures of GO were destroyed during the oxidation process for access to the functional groups, such as −COOH, −C=O, and −O−. A similar result was reported in a previous work [40]. Moreover, PANI/GO composites (Figure 6b) displayed an irregular, multilayered morphology, largely due to the wrapping of PANI on/between the GO sheets to form a flossy structure. As a comparison, a perfect crystalline structure and no lattice distortions were observed in the PANI/M-GO composites, as shown in Figure 6c, which may be expected to bring high electron collection and transfer properties when used as electrode materials.

#### 3.4.2. SEM Observations

**Figure 7:**
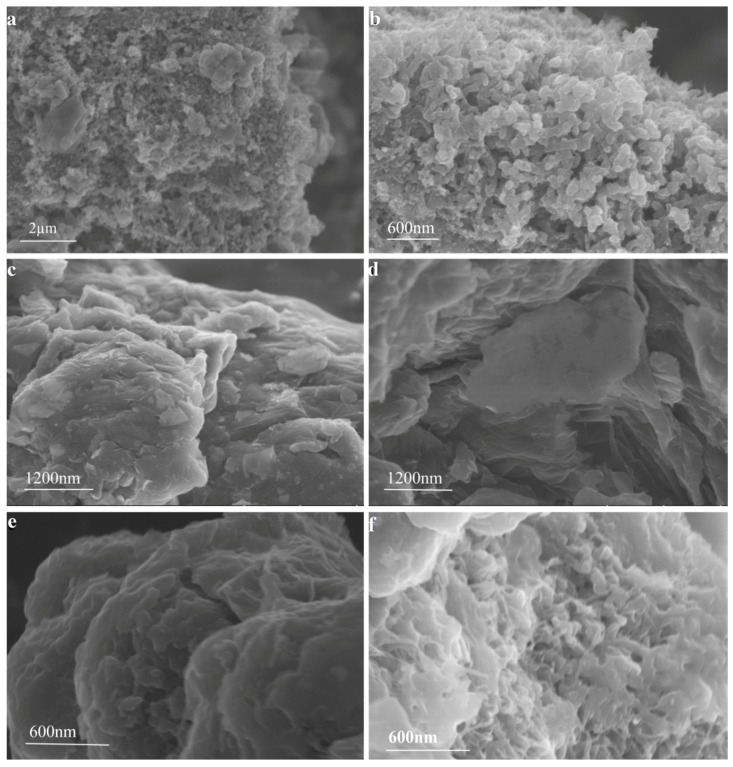
SEM images of (**a**,**b**) PANI; (**c**) GO; (**d**) M-GO; (**e**) PANI/GO; and (**f**) PANI/M-GO composites.

Figure 7 shows SEM images of PANI, GO, M-GO, and PANI/GO composites. Apparently, pure PANI (Figure 7a,b) has a compact fibrous structure with a narrow size distribution in both diameter and length. GO (Figure 7c) was observed to have a flaky texture, prone to twinning and aggregation. In comparison with GO, M-GO (Figure 7d) exhibited a much better dispersion. Furthermore, M-GO behaved as a rather highly ordered layered structure, indicating that microwave pretreatment of graphite imparted an enhanced specific surface area to M-GO, which could facilitate the bonding between PANI and GO. To further identify the positive role of microwave pretreatment of graphite in the formation of PANI/GO composites, the morphology of PANI/GO and PANI/M-GO composites was compared, as shown in Figure 7e,f, respectively. It is widely accepted that in polymerization, graphene oxide acts as a template, and the PANI is prone to grow orderly on the surface of graphene oxide largely due to the π-π interaction, electrostatic forces, and the hydrogen-bonding between graphene oxide and PANI [33]. It can be observed here that the PANI nanofibers were randomly distributed on the surface and/or between the GO sheets (Figure 7e). However, the PANI/M-GO composites exhibited a relatively compact network structure with well-arranged PANI nanofibers on the M-GO sheets (Figure 7f). The close bonding between PANI and GO was expected to collect electrons from an external circuit to the GO layers and then transfer them to the PANI chains.

#### 3.4.3. FT-IR Spectra

**Figure 8:**
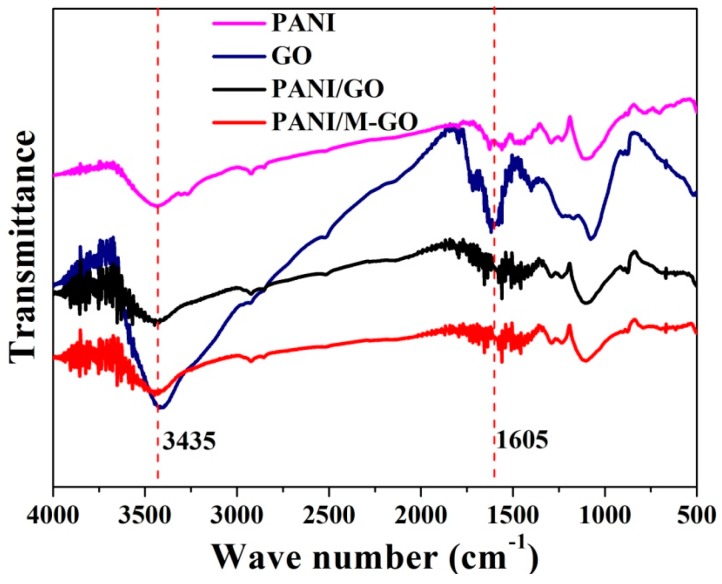
Fourier-transformed infrared spectroscopy (FT-IR) spectra of PANI, GO, PANI/GO, and PANI/M-GO composites.

The FT-IR spectra of the PANI, GO, PANI/GO, and PANI/M-GO composites are shown in Figure 8. For pure PANI, a broad band above 2000 cm^−1^ was mainly attributed to the presence of free charge carriers in the PANI emeraldine salt. The peaks at 1560 and 1483 cm^−1^ corresponded to the C=C stretching modes of the benzenoid ring and C=N stretching of the quinoid ring, respectively, which represented its electrical conductive properties. The peaks at 1293 and 1112 cm^−1^ were characteristic of C–N and C=N stretching vibrations, respectively [41]. A shoulder at 1090 cm^−1^ was assigned to C–H in-plane bending on the para-disubstituted ring. For GO, the absorption peaks at 3390, 1729, and 1398–1077 cm^−1^ were attributed to the –OH, C=O, and C–O in COH/COC (epoxy) functional groups, respectively [42]. As commonly observed in PANI/GO and PANI/M-GO composites, the characteristic peaks of PANI and GO were observed, which confirmed the successful synthesis of PANI/GO composites. However, there were some minor differences between the FT-IR spectra of all the samples. Specifically, the stretching vibration of C=C in PANI and the benzenoid ring were slightly red-shifted to 1568 and 1489 cm^−1^ in comparison with the bands of pure PANI as a function of π–π interactions and hydrogen bonding between the doping graphene oxide sheets and the PANI backbone [43]. In addition, the slight increase in the intensity of bands at 3435 and 1605 cm^−1^ in composites might further identify the combination of PANI and GO.

#### 3.4.4. XRD Analysis

**Figure 9:**
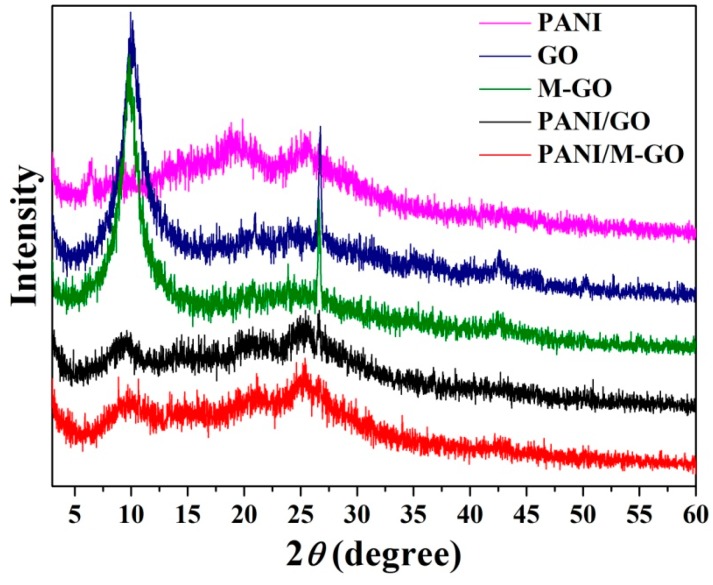
X-ray diffraction (XRD) patterns of PANI, GO, M-GO, PANI/GO, and PANI/M-GO composites.

The XRD patterns of the PANI, GO, M-GO, PANI/GO, and PANI/M-GO composites are depicted in Figure 9. For PANI, the crystalline peaks appeared at 2θ = 19.4° and 25.4°, corresponding to the (020) and (200) crystal planes of PANI in its emeraldine salt state [44]. GO exhibited an intense, sharp peak centered at 2θ = 10.12°, corresponding to the (001) interplanar spacing of 0.873 nm, as calculated by Bragg’s law:

(2) $$d=\frac{n\lambda }{2\sin\theta }$$

In Equation (2), *n* is chosen as 1, λ is 0.154 nm for the wavelength of Cu-Kα radiation, and θ is the Bragg angle. This interplanar spacing was different from that of the raw graphite (0.335 nm), indicating the exfoliation of graphite to form GO [45].

For M-GO, the (001) diffraction peak gradually shifted to 9.90°, and the interplanar spacing (0.892 nm) was slightly higher than that of GO, which is consistent with the above results. The X-ray data of the PANI/GO and PANI/M-GO composites presented crystalline peaks similar to those of PANI, implying that no additional crystalline order has been introduced into the composites. Moreover, the diffraction peak of GO and M-GO almost disappeared, indicating that the GO and M-GO had no serious aggregation, fully interacted with the PANI molecules, and was completely covered by PANI particles to produce layered composites.

#### 3.4.5. TG Analysis

**Figure 10:**
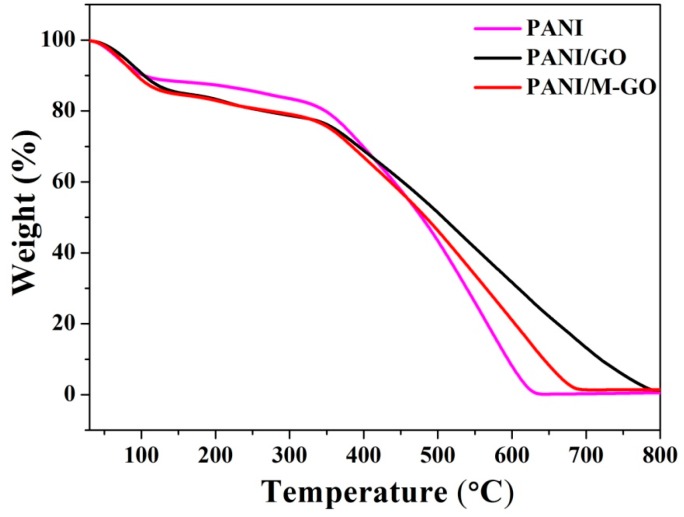
Thermogravimetric (TG) curves of PANI, PANI/GO, and PANI/M-GO composites.

The TG curves of the PANI, PANI/GO, and PANI/M-GO composites are given in Figure 10. All the samples showed a gradual weight loss at approximately 100 °C, mainly due to the evaporation of the absorbed water. The PANI sample displayed an accelerated weight loss at 350–600 °C due to the pyrolysis of the polymer, similar to the previously reported results [46]. Furthermore, the thermal stability of the PANI/GO and PANI/M-GO composites was compared. The PANI/M-GO composites tended to decompose slightly earlier than PANI/GO composites within the temperature range of 350–800 °C. The decreased thermal stability of the PANI/M-GO composites is presumably due to the fact that microwave treatment of graphite imparted a relatively ordered layered structure to the GO and in turn facilitated the dispersion of the resulting PANI/GO composites, thus eventually leading to the somewhat quick pyrolysis of the as-prepared PANI/M-GO composites.

## 4. Conclusions

Treatment of graphite with microwave radiation was proposed and demonstrated to improve the electrical conductivity and electrochemical properties of PANI/GO composites synthesized by a soft chemical method. It was evident that a homogeneous composite was achieved by the presence of PANI particles distributed on the surface of or between the GO sheets. The electrical conductivity, specific capacitance, and cycling stability of the PANI/GO composites were enhanced due to the microwave treatment of raw graphite, particularly when the An/GO mass ratio was 25:1. The above results support the conclusion that microwave treatment of raw graphite exerted a pronounced effect on the electrical conductivity and the electrochemical properties of PANI/GO composites. The composites showed good properties for potential application in supercapacitors or other power source system.
